# Influence of Pathological Anatomy upon Medicine, from the Time of Morgagni to the Present Day

**Published:** 1838-10

**Authors:** 


### BRITISH AND FOREIGN MEDICAL REVIEW,

FOR OCTOBER, 1838.

### PART FIRST.

### Analytical and Critical Reviews.

### Art. I.

1. *Influence de l'Anatomie Pathologique sur la Médecine, depuis Morgagni jusqu'à nos Jours.* Par Risueno D'Amador, Professeur à la Faculté de Médecine de Montpellier, &c. &c. 4to. pp. 181. (*Memoires de l'Academie de Médecine*, tome vi.—*Paris*, 1837.)
*Influence of Pathological Anatomy upon Medicine, from the time of Morgagni to the present day.* By Risueno D'Amador, Professor, &c. &c.

2. *Mémoire en Réponse à cette Question: " Quelle a été l'Influence de l'Anatomie Pathologique sur la Médecine, depuis Morgagni jusqu'à nos Jours?"* Par Constant Saucerotte, Docteur en Médecine, &c. &c.—4to. pp. 111. (*Ib.*)
*Memoir in Reply to the Question: " What has been the Influence of Pathological Anatomy upon Medicine, from the time of Morgagni to the present Day?"* By Constant Saucerotte, m.d. &c.

The two dissertations the titles of which are above transcribed, were submitted to the Royal Academy of Medicine in the *Concours* of 1836 for the prize founded by the late Baron Portal, for the encouragement of pathological anatomy. One of these, (that written by M. Saucerotte,) received the approbation of the academy, while the other obtained the prize. This last is a production of very considerable merit. The question proposed by the academy, was to determine the influence of pathological anatomy upon the science of medicine from the time of Morgagni to the present day. M. D'Amador in replying to this question, divides his subject into two parts, the first of which is devoted to the consideration of certain laws laid down as those under which he conceives the development of morbid anatomy to have proceeded; the second, to the solution of what he terms the three anatomico-pathological problems in their application to the science of disease. These problems respectively relate to the organic change, to the symptoms, and to the causes, as will hereafter be seen. From the mere enunciation of the primary divisions of this treatise, we are at once led to expect an attempt at something like precision in the general conduct of the work, an expectation which a more extended examination of its pages confirms; and, indeed, a closer attention to the principles of the inductive philosophy will be found throughout than is customary in writings devoted to the elucidation of medical science.

It has ever been found advantageous to take, from time to time, a retrospect of the progress made in the acquisition of knowledge,—to pause, as it were, on some eminence gained, and to contemplate at once from this vantage-ground the steps successively attained, and the vast field which lies yet untrodden before us. In the darker periods of the science of medicine, the several theories which successively sprung up, and, for a time, held possession of the minds of those who were but too readily disposed to follow the leading of a great name, seem to have conduced to this important end; while the more modern eras have been similarly marked by the researches of observers devoting their time and talents, in a more cautious spirit of observation, to the elucidation of parts of the great whole, leaving it to some future master-mind, which may prove the Cuvier or Jussieu of our science, to combine all these into a complete and harmonious system.

The leading principle, however, "*l'idée mère*," that which gravitation is to astronomy, or the doctrines of proportion to chemistry,—yet remains to be developed in the medical sciences; and, until the *special* laws of life and organization shall have been ascertained, and the combination of them into more *general* laws effected, we must still remain in the present unsatisfactory state of hesitation and doubt, with respect to numerous questions of vital import to the true comprehension of the phenomena of health and disease. The essay of M. D'Amador contains materials which, considered in this point of view, are applicable to the illustration, not only of pathological anatomy, but also of the science of medicine taken as a whole.

The first part of the essay, is devoted to the elucidation of what the author terms *the Laws of Development of Pathological Anatomy*. In the attempt to establish these laws, M. D'Amador traces the progress of pathology in each of the leading systems which have at various times obtained the ascendant. In the earliest periods to which our records extend, when, amidst much uncertainty, some important truths had already been appreciated, the germ of these several systems may be detected; but with the gradual increase of knowledge came also the attempts at explanation of all known phenomena upon some general and exclusive principle, and hence sprung up the successive and rival codes of the vital, humoral, and organic pathologies, each, in its turn, professing to trace every aberration from the physiological state to an alteration in the solids, in the fluids, or in the vital forces.

The progressive advance of these systems, however, does not appear to have been made in accordance with any general rule, as, from the more gross to the more refined material agent, and thence again to the more subtle agencies of the vital system; but seems rather to have proceeded in a series of oscillatory gradations, to have vacillated, as it were, between the extremes, each successive period being enriched by the acquirements of that which had preceded it. It is thus that the doctrines of the schools, having been in succession exclusively vital, and exclusively humoral, became at a subsequent period exclusively organic, until at length it has been again acknowledged that the fluids must also challenge for themselves a due consideration in a true and extended system of pathology; and, more recently, that the vital influence—the influence arising from the presence of the principle of life, and under the regulation of the laws which govern the action of that principle, must pervade the whole. There is this especial difference, however, in the code of pathology professed by the modern, or, as it has been termed, the eclectic school,—that, at the same time that it recognizes the fluids and vital powers as necessarily bearing an important part in the theory of disease, the alterations in the tissues and organs constituting the solid parts of the body are not overlooked nor disregarded.

The first law of development proposed by M. D'Amador (although, according to our view of the matter, the proposition contains the statement of a *fact* not a *law*,) is, that *Pathological Anatomy, as well as the other Elements of Medical Science, already existed in the germ at the commencement of Medicine.* In illustration of this principle, which will scarcely be questioned if we are to receive the term medicine as intended to apply to the science, the author instances the description of the development and progress of tubercles by Hippocrates, and the circumstance that Aretæus was aware that paralysis occurs on the side opposite to that on which the brain is affected; and he might have referred to the still earlier period, in which the sacred historian so admirably describes the distinctive characters of the Jewish leprosy and some allied forms of cutaneous disease. But the epoch of pathological anatomy can scarcely be said to have really commenced until the time of Morgagni, and, in tracing the history of medicine, we can scarcely date its influence much anterior to that period. During the long period in which the Galenical doctrines of the four humours held undisputed sway, medicine, as a science, remained absolutely stationary, and it was not until the examination of diseased structure was generally had recourse to that it can be said to have made any real progress. At first, however, it was only the more extraordinary cases which were thought worthy of investigation or description, and with respect to the alterations produced by diseases of frequent occurrence, it was, as M. Saucerotte remarks, much too common an affair to require looking into. Hence we find Sylvius, Bartholin, Renealmus, Tulpius, Panarolus, and many other writers of that period detailing cases of disease and morbid changes, bearing about the same relation to the descriptions of the modern pathology as the romances of the troubadours, and the fairy tales of the nursery, do to a correct historical or biographical memoir. It is to Morgagni that we especially owe the impulse to a more rational employment of this mode of studying disease, and his immortal work *De Sedibus et Causis Morborum* will ever remain a practical illustration of the important truth, that the more frequent a disease, the more necessary it is that its phenomena should be carefully investigated and thoroughly understood. Without enquiry into those alterations of structure which take place during the course of morbid actions, or are otherwise connected with them, it is impossible to comprehend the symptoms by which they are indicated, and the mode of operation of the causes to which they owe their rise. In reference, therefore, to the organic changes which takes place internally, and to the rationale of disease consequent thereon, pathological anatomy may be said, in the somewhat obscure terms of the second law, to represent in the history of medicine "*le developpement interne, reflechi, et logique de la science.*"

The third law proposed by M. D'Amador requires a rather more extended consideration. It professes, as we understand it, that *pathological anatomy in the course of its own progress has given a character* ("*a revêtu les formes*") *to the general systems of medicine which have successively prevailed.* There seems, however, to be some ambiguity in the enunciation of this law, since many of the observations which follow —to which, we may remark, the facts of the science themselves accord— imply just the reverse, viz. that pathological anatomy has in the course of its progress assumed to itself the characters of the systems which have successively prevailed.

At the time when surgical or external diseases became the especial objects of attention, the chief application of pathological anatomy was directed towards the exposition of changes taking place in the organization of the exterior organs or parts of the body; and subsequently when extended to the elucidation of internal diseases, the same spirit of adaptation to existing views would seem to have prevailed. Accordingly, we find Bonetus and the writers of that period, who were occupied rather in the endeavour to account for the fatal termination of a malady than in tracing its rise and progress, seeking the cause of death in the changes discoverable in the internal organs, and recognizing as such, alterations of structure very inadequate to the effect, provided they were of sufficient magnitude to attract attention.

It is sufficiently evident that the long exclusion of changes in the fluids, and in the distribution of the more subtle agents connected with the phenomena of life from their due place in the doctrines of disease, has been mainly owing to the exclusive direction given by the study of pathological anatomy towards the solids; in other words, that the predominant system of a purely organic doctrine of disease has been invested with an importance by the cultivation of morbid anatomy to which, considered as an exclusive system, it is by no means entitled. At the same time, it seems clear that an impulse was given to the cultivation of pathological anatomy by the existing doctrines of solidism, rather than that these doctrines took their rise from the attention given to the study of diseased structure. This however, after all, is a question upon which there may be probably some difference of opinion, and at the same time one which, considered apart from the terms of the law of development laid down by M. D'Amador, is now of little importance. A more correct expression of this law, or fact, as it appears to us, is, that pathological anatomy in its progressive development has borne an intimate relation to the doctrines of pathology which have successively prevailed. However this may be, there can be no doubt that the researches of Morgagni in the first place, and subsequently those of Carmichael Smyth, Pinel, Bichat, Laennec, Dr. Abercrombie, M. Broussais, and others, which have contributed so much towards a correct knowledge of the changes induced by disease in the organization of the human body, have in this respect, by their very importance, tended to impede the study of those changes in the condition of the fluids, or in the action of the vital forces, which are of at least equal importance in a true system of pathology. Some of the errors thus resulting from the exclusive attention paid to changes of structure in the solids are well pointed out by M. Saucerotte.

"Not only has this assiduous investigation into those diseases which, as it was thought, admitted of explanation from certain structural lesions, caused the consideration of the vital phenomena to which they are subordinate to be neglected; not only has it accredited the false idea that every functional disturbance is necessarily accompanied by sensible material changes; but it has also caused us to lose sight of the constitutional origin of a host of local affections, themselves only the crises of diseases; it has turned aside our attention from those epidemic influences which despotically govern disease, rendering necessary the most different modes of treatment when the organic lesions are the same, (facts well adapted to show with what distrust the opinion that anatomical observations should form the sole basis of pathology is to be received): finally, since morbid anatomy has been limited to the study of alterations of the solids, it has contributed to confirm the absolute dominion of that exclusive solidism, which leaves without its narrow limits so many unexplained facts." (p. 544.)

The great error of the organic pathology would however seem to be, that the distinction between the seat and the cause of disease has not been sufficiently borne in mind. It is thus that the alterations in the organization which, at one time, were regarded as indicating the cause of death, were subsequently considered as, in their initial state, constituting the cause of the disease. Really, however, these changes are in themselves no more than links in the chain of diseased action which is, as it were, thereby localized, but the original cause of which is to be sought elsewhere. It is thus also, that the luminous views of Bichat, and his predecessors, Carmichael Smyth and Pinel, have been distorted by the one-sided speculations of more recent writers, until the last finish was put to the organic pathology by the wild reveries of M. Broussais, who, with the most perverse ingenuity, has contrived not only to give a local organic cause to almost every disease, but actually, in a great measure, to limit the seat of this cause to a part of one particular system of the human frame, seeing in a *gastro-enteritis* the fertile origin of every disease which flesh is heir to.

M. D'Amador correctly observes, that the progress of pathological anatomy has, for some years, taken a direction towards the humoral pathology, and traces this change to the publication of the *Clinique Médicale* of M. Andral in the year 1825. Had M. D'Amador been as well acquainted with the writings of British authors, as he has shown himself to be with those of his own countrymen, he would not only have discovered similar traces of this change for some time before the appearance of the work of M. Andral, but also, that the pathology of the British school has never been so exclusively organic as that of the French; and, putting out of the question John Hunter, in whose writings will be found many passages tending to show that that master of our science has anticipated much of what is now brought forward by M. Andral and his followers, he would also have discovered that Drs. Prout, Burne, Carswell, Sir James Clark, and many others, of whose existence even he seems not to be aware, have done more towards the elucidation of these great questions than even the great chief of the *école eclectique* himself, to whose writings he attributes so powerful an effect. We have said that the origin of this change is referred by the French pathologists to the year 1825, and this period seems at least that from which we must date its progress amongst themselves. In 1829, we find M. Cruveilhier saying, "the more we study diseases, the more we seek to discover their immediate seat, the more are we led to think that the liquids are the vehicle of a great number of morbid causes, that a complete system of pathology should embrace the lesions of both liquids and solids." These opinions, which we are told were at that time confined to the brighter geniuses (*esprits élevés*,) were subsequently spread throughout all ranks of the profession, and thus, as M. D'Amador observes, the insufficiency of the solids has led to the study of the liquids, as the insufficiency of the seat has led to the study of the cause, and the insufficiency of local disease to the study of general disease.

The recognition of the agencies of vital forces soon followed, or was rather perhaps contemporaneous with the progress of the humoral code, and is equally attributed by M. D'Amador to MM. Andral, Lobstein, and Cruveilhier.

"We may say, then, that by a long and painful path, the science of pathological anatomy has at length itself attained to the confirmation of the aphorism of the school of Hippocrates—'that we must consider in man not only the solids or containing parts, and also the liquids their contents, but above all the active forces, or those which give rise to motion.' Organic science commencing with the solids, concludes with vitalism, that is to say, with the Hippocratic doctrine properly comprehended; whilst, on the other hand, the school of Hippocrates commencing in vitalism, concludes in the course of ages by associating itself to the science of organic pathology: a fatal circle run by these doctrines, which by their ultimate concurrence prove at once both their individual insufficiency and their reciprocal dependence. For be it remarked that, at all times and in all cases, the fact—the practice—that which in the mass is called the history, commences by preceding the theory." (p. 333.)

These progressive steps which are, in the first place, brought forward as matters of historical demonstration, are then shown to be in accordance with what might have been reasonably anticipated. The organic pathology, being that which, by the more evident alterations of volume and consistence, the increase or diminution of organs, their hypertrophy or atrophy, reveals itself at the first glance when attention is directed to what is passing in the interior of the body, would naturally be the forerunner of those systems in which the more subtle though no less evident changes in the fluids or vital forces, are allowed their due place. But when the more refined operations of the body, whether in the state of health or disease are scrutinized into; when the molecular actions are investigated, the insufficiency of the solids to account for the phenomena, whether physiological or pathological, becomes instantly apparent. It is then that the blood, which Bordeu, in the spirit of the organic pathology, had asserted to be nothing more than liquid flesh (*la chair coulante*,) is recognized as being possessed of innate powers, and as materially influencing, and not unfrequently causing, by changes in its component parts, the series of diseased actions. The study of alterations taking place whether in the blood, or in the other fluids of the system, is but a more refined species of pathological anatomy; the mechanical process of the scalpel being here inadequate, the more refined processes of chemistry are called to our aid, and variations in the nature of the fluids which had otherwise escaped our notice are thus detected. It is obvious, that, whether these variations are to be considered as proximate or ultimate links in the chain of disease, they are deserving of our most attentive consideration. The enquirer into the pathology of typhoid fevers, for instance, who should content himself with merely ascertaining that congestions in the capillaries of the gastro-enteric mucous membrane are present in certain forms, while in others an injected state of the arachnoid tissue may be detected, overlooking the loose dissolved consistence and black colour of the blood, and the changes which take place in the proportion and qualities of the urine, perspiration, and other excreted fluids, will form a very imperfect idea of the nature of this family of diseases. And yet, not only are these fevers localized in one or other of the sites specified by high authorities, but their very essence is, on the one hand, asserted to be cerebral inflammation, on the other a gastro-enteritis.

It is not, however, our purpose here to refute these exclusive doctrines of the organic pathology. We are desirous merely of enforcing what M. D'Amador throughout his treatise ably points out, that the province of pathological anatomy is rather to detect the changes which have taken place, in the course of morbid actions, in the structure of the tissues and organs of the body, to demonstrate from such changes the seat of local alterations, to investigate their nature, and to determine their relation with the general progress of the disease and its symptoms during life; but that with respect to the nature of the proximate cause, the essence of the disease, the *primum mobile* of the organic aberration from the state of perfect health, it reveals absolutely nothing. Pathological anatomy will teach us that the lungs, for instance, may have been the particular organ in which the diseased action has been going on; that the changes thus induced are consolidation of texture, a distended state of the capillaries of the mucous membrane, or tubercular deposition in the bronchial tubes and cells. It may show that inflammation, or tubercular disease has existed therein; it may point out that the alterations of texture are in strict accordance with the physical signs afforded during life by auscultation and percussion; but to determine the peculiar aberration from the state of health which constitutes inflammation, tubercle, rheumatism, or any other morbid process, is beyond its powers and foreign to its true objects. The irregularity in the molecular actions which constitutes the essence of these maladies, may probably for ever elude our search, though it is the aim of the modern pathology to seek to appreciate this.

The theories of disease have in succession been explained, according to the vital and humoral systems, by the fanciful doctrines of animal spirits, of the four humours, of the presiding Archæus of Stahl, and the more rational humoralism of Boerhaave, Van Swieten, and Gaubius; according to the organic system of Hoffman and his followers, by alterations taking place in the actions of the solids, whether accompanied or not by changes of structure. "Where," asks M. Saucerotte, "was the concealed flaw which paralysed the productions of so many eminent men? It is, that from the consideration of the exterior phenomena of diseases, they proceeded at once to the consideration of the primitive forces impressed upon the organism, without stopping at the organic lesions by which they are necessarily connected; the intermediate link without which the two extremities of the chain could never have been united." (p. 501.) Between the disease itself, then, and the symptoms by which it is manifested, there is a link, or series of links, wanting, and it is in the filling up of this chasm that pathological anatomy has, according to M. D'Amador, followed the same course as the theories which have successively had the ascendant.

The fourth law declares that "*pathological anatomy, in each of its systematic transformations, has exerted a powerful influence upon the other branches of medical science, and has favoured their progressive development.*" It is unnecessary here to enter at any length upon the investigation of this law. It is sufficient to refer to what has been already said, to show the progress made in the doctrines of disease since the more assiduous cultivation of morbid anatomy. The several systems of the organic, humoral, and vital pathologies have been successively preferred, and the necessity of their combination in an extended and comprehensive theory of disease recognized through the insufficiency of any one to afford a satisfactory explanation of the phenomena. Among the other results springing from the investigation of organic changes, M. D'Amador enumerates the general improvement of the spirit of medical science, the impulse given to the methods of discovering and demonstrating medical facts, the improvement of organic chemistry and of comparative anatomy, &c. Our limits will not permit us to enter upon the illustration of these remarks supplied by our authors.

The fifth law is, that "*pathological anatomy concludes by exaggerating its own principle, thus constituting itself into an exclusive system.*" This is evidently the natural tendency of its cultivation upon a system of pathology essentially and exclusively organic, and therefore, as M. D'Amador observes, this law is but the abridged formulary of the history of medicine. The subsequent pages of this part of the treatise are accordingly devoted to a rapid summary of the progress of pathological anatomy itself. In this sketch we cannot follow our author, neither is it necessary for the due comprehension of the subject that we should make the attempt. It may however be stated, that after referring to Hippocrates and Aretæus, as having shown a remarkable knowledge of some diseased processes, the principal writers mentioned as contributing in an especial manner towards the progressive advance of this branch of medical science are Bonet, Morgagni, Bichat, and Andral, each of whom, more especially the three last, are to be considered as having constituted by their observations and writings a new era in this science. Here, as throughout, M. D'Amador appears to be lamentably ignorant of English and German authors. He enumerates, however, the worthies of the "*école anglaise*," who follow, according to him, in the steps of Morgagni; and these are J. Hunter and Home (!) as the founders of the school, and *Sthear* and *Abernety* (sic) as its most recent cultivators! In the identification of Abernethy we have not much difficulty, although we may demur as to his claims to the place assigned him; but, as to who Sthear may be, we confess our utter ignorance. This school, however, he admits is rather than Morgagni to be considered as the forerunner of Bichat, and indeed that it almost anticipated him; and he might have added, had he been really acquainted with the writings of J. Hunter,—Andral, also. We cannot resist transcribing the following exquisite morsel in which the author of the *Anatomie Générale* is introduced: we will not lessen its force by attempting to clothe it in an English dress. "Or, trente ans après qu'il fut achevé, (the elevation of pathological anatomy into a science by

Morgagni,) c'est un Français, c'est Bichat, dont le génie trouve ce que le génie de tous les médecins anatomistes n'a pu encore trouver. C'est Bichat qui enseigne à l'Europe entière les principes et les lois des faits, et les regards étonnés se fixent sur l'auteur de l'*Anatomie Générale*, qui réalise si bien la prophétie qu'en avait portée Sandifort." (p. 387.)

Proceed we now to the second part of the essay, which professes to be a solution of the three anatomico-pathological problems in their application to the science of diseases. These problems are: 1st, that of the organic change; 2d, the relations of this change to the symptoms; 3d, that of the cause or of the diathesis.

"Every disease when fully formed," says M. D'Amador, "consists of external phenomena called symptoms, of organic changes, and of modifications in the vital forces which properly constitutes the affection itself, the morbid condition or diathesis." It is the middle term, the organic alterations, which forms the subject of the first enquiry. The two leading sections of the ancient school, the dogmatists and the empirics, were both deficient in this point, and the latter of these was content with the study of the symptoms alone. The former attempted to investigate the morbid condition, as well as the symptoms to which this condition ultimately gave rise; but the connexion between these two could not then be detected, and it is the school of pathological anatomy which has supplied the link wanting by the gradual development of the intermediate organic changes. The progress made by this school has been in accordance with the laws of induction; it has traced the relations existing between the external manifestations of disease and the organic alterations which have arisen in its course, and though subsequently the principle was carried out too far, yet when accumulated observation has demonstrated that residual phenomena exist, for the explanation of which the leading idea of organic change is insufficient, the modern school of pathology seeks a more general law under which their phenomena may be comprehended, and is thus led to discriminate between the organic changes as the effects of diseased actions, localized in the organs and tissues, and the actions themselves. This then is the province of pathological anatomy, not to form a system of pathology, but to develope the alterations arising in the tissues and organs of the body in the course of disease, and to determine the laws of their formation. "Le problème médical," says M. D'Amador, "a souvent changé de face." It has been stated in these terms,—A disease being given to find the remedy:—this is the purely practical view, and will recommend itself to the mere utilitarian. Pinel changed the question,—A disease being given to give it a character and to determine its place in a nosological chart: this is the view of the systematist. The pathological anatomist, again, says,—A disease being given to discover its seat and to determine the organic change in which it consists:—this is the view of the man of science, but it is imperfect and insufficient, inasmuch as, although a correct pathology always contemplates the three elements of disease, the external symptoms, the organic change, and the cause from which these spring, yet the existence of each of these elements is not necessarily implied in every disease; it is the last only, the cause, which is essential and without which the other elements cannot exist.

It has been observed, that it is the province of pathological anatomy to determine the seat of diseased action; and it is instructive to trace the progressive refinement which has been attained in effecting this object. In the earlier stages of this enquiry, the question involved little more than the organ affected: gradually, however, it was perceived that the various parts of which the organ consisted were liable to peculiar changes; as, for instance, the air-tubes of the lungs to catarrhal discharges, the cavities of the pleura to serous accumulations and adhesions, &c. The study of the membranes by Pinel, Carmichael Smyth, and Bichat led to the generalization of these facts; and the law was established, that catarrhal affections appertain to the mucous membranes especially, and effusion of serum to the serous membranes. It may be readily perceived that there are reasons for this in the general conformation and distribution of these tissues; but the occult causes, which must be sought in the molecular structure of the parts, have not as yet been revealed. A necessary result of this progressive advance in the investigation of morbid products has been a material change in the mode of contemplating disease. Many affections, which were formerly separated from each other, have been ascertained to be mere modifications of the same morbid condition; while others, again, have been separated from those with which they were formerly confounded, and distinguished by appropriate signs. The precision at which we have thus arrived in diseases of the chest may be adduced as affording illustration of the preceding remarks. The external signs of cough, expectoration, dyspnœa, various degrees of pain, &c., are common to almost all; and in many instances are altogether insufficient for the distinguishing of affections very different in their nature. Pleurisy, pneumonia, peripneumonia notha, catarrh, hæmoptoe, phthisis, asthma, dyspnœa, and hydrothorax constitute the list of pulmonary diseases formerly received; and how ill these affections were defined, and how utterly impracticable it was to distinguish one from another, need not here be insisted upon. Pathological anatomy, however, establishes some of these upon just grounds; indicates the existence of many which had not been previously recognized, or had been confounded under the vague terms of asthma and dyspnœa; and points out those differences in the organic changes which afford distinguishing characteristics: but it is a matter of vast importance to be enabled to ascertain these differences in the diseased state during life. New methods of investigation are required; and it was reserved for the admirable sagacity of Laennec, working still upon the foundation of pathological anatomy, to devise and perfect those methods of auscultation and percussion which leave in this department little to be desired.

In proportion to the advance of pathological anatomy, then, are we enabled to connect particular symptoms with particular organic lesions, and thus to ascertain the *proximate causes* of disease. In the same proportion, it appears to us, will it be found that the term *disease* will naturally be applied to the abnormal condition thus recognized, and not to the mere external manifestations of it. The improvements in diagnosis to which we have just alluded have effected this in a very remarkable degree as to complaints of the chest; and, when the diseases of other parts of the system shall be as well understood, we venture to predict that the Cullenian definitions, admirable as they are if looked upon simply as *descriptions* of groups of phenomena, will no longer be regarded as characterizing the morbid states whose cognizable effects they so graphically delineate. Let us draw an illustration of our meaning from mechanical science. A steam-engine may be compared in many respects to the living body: its different parts are *organized* to perform different functions, all tending to one end; and, for the maintenance of these functions, it is dependent upon the continued supply of external *stimuli*,—fuel to the fire, water to the boiler, and cold to the condenser; moreover, it possesses, by means of its *governor*, a certain degree of power of self-adaptation to changes in its external conditions. Now, from the excessive or deficient supply of these, irregularities in the operations of the machine will manifest themselves. Thus, an additional quantity of fuel may have been laid on the fire, or the water in the boiler may be low so that steam is generated too rapidly, or the quantity of work required may be suddenly diminished. The effect of either of these causes will be the same, acceleration of the motions of the engine. The latter, then, according to the old method of describing diseases, would be the sole account given of the disorder of our machine, although it might have arisen from either one of three different causes. It is obvious that no engineer would rest satisfied with such a symptom, but would, before attempting to apply his remedy, set himself to enquire what is the cause of the irregularity; on what change of the conditions of the machine it is dependent. This, however, is a very simple case. We will push our analogy a little further. We will suppose that, after violent irregularity of action, the machine suddenly stops. The engineer will, of course, do his best to restore its motion, by the alteration of the conditions on which it depends; but, if unsuccessful, he will search into the internal structure of his machine, with the view of discovering the cause of his failure. There he may find the displacement of a valve, the fracture of a rod, or the abrasion of the packing by which the piston is adapted to the cylinder, which may in some instances have been the original *cause* of the irregularity of action which preceded the cessation, or in others it may have been itself the *result* of an irregularity dependent upon some of the causes formerly mentioned. We hope that this illustration may set in an evident light the relation between pathological anatomy, pathology, and practical medicine.

These observations naturally lead us to the consideration of the second problem,—that of the relations existing between the organic change and the external symptoms by which it is accompanied.

In a perfect symptomatology, says M. D'Amador, this problem should be proposed as follows: 1. To determine, by the symptoms, the seat and nature of the organic change; 2. To explain, by the seat and nature of the organic change, the symptoms observed. The first question should be resolved during life, the second can be resolved only after death. The complete solution of these questions, however, implies, first, that every disease shall leave such marks of its progress as are appreciable to the senses, and that these alterations shall be always of the same nature; secondly, that the changes induced by death shall be sufficiently distinct from those produced by the disease; and, thirdly, that these last shall never disappear with the cessation of life, to give place to phenomena purely cadaveric. It is not necessary here to point out that, in the present state of our knowledge, these objects are unattainable; but that much may be effected by careful observation and study, in unravelling the intricacies which involve this question, is manifest from what has been already said in respect of diseases of the chest. That these difficulties, however, are altogether insurmountable, as M. D'Amador seems to apprehend,—at least in respect to many of the instances which he cites,—we are by no means prepared to admit. It is not because no proportion is found to exist between the extent of certain morbid alterations and the symptoms by which we are at this time accustomed to seek for them, that we are to draw the conclusion that such a proportion may not hereafter be discovered. Future researches may open to us other methods of investigation which shall sensibly indicate these changes in proportion to their extent, although, perhaps, the relative importance of the organ affected, or its want of sensibility, or its situation in the innermost recesses of the body, may prevent the manifestation of the changes of structure which it may be undergoing, in a corresponding amount of physical suffering, or other readily recognized signs. The pain or inconvenience experienced by a person labouring under very extensive disorganization of the lungs, for example, is sometimes comparatively trifling, and in slighter cases may be, and frequently is, altogether overlooked; and in such instances we cannot doubt that the true nature of the disease, as well as its extent, would formerly have long escaped detection. But, since the introduction of new methods of investigation, percussion and auscultation, the most intimate relation between the organic changes and the symptoms by which they are accompanied is found to exist. Dr. Bright's admirable researches upon disease of the kidney in connexion with an albuminous state of the urine, followed up and rendered more definite in their application by Drs. Christison, J. C. Gregory, and Osborne, afford another excellent illustration of the remarks we have just made. It may possibly be objected that the signs of disease afforded by these methods of exploration are not symptoms in the usual acceptation of the term; but to us the dull sound beneath the clavicles elicited on percussion, or differences in the respiratory murmur in the same region, perceived by the application of the ear, are as clearly symptoms of disease as the pain or tumefaction, detected only on close and accurate manual examination, of parts deeply seated in the abdomen or pelvis. If, however, we are to understand by the term symptoms, merely those evident signs which are of such magnitude as to attract the notice of the sufferer, or to give rise to much personal inconvenience, then it must be granted that no relation can ever exist between the internal change and the symptoms attending it; and, further, that, thus limited, the study of symptoms, so far from aiding in the discovery of disease, reveals nothing but a tissue of contradiction and fallacy.

It would be an instructive task to examine the instances brought forwards by the author, as well as numerous others which might be adduced in illustration of these observations; but we are reminded that another problem, that of the causes or diatheses, yet remains for consideration.

This problem, says M. D'Amador, embraces three principal questions, to be resolved by pathological anatomy: 1, To prove, by pathological anatomy, the existence of diatheses or general conditions, tending to disease; 2, to show the influence of these as occasional causes in the production of organic changes; 3, to study the influence of inflammation, first considered as a diathesis, and subsequently as an occasional cause of change of structure. To these might have been added with advantage the study upon similar principles of the tubercular, the carcinomatous, and other no less marked diatheses, which, especially the tubercular, are of equal importance with that of inflammation.

These questions, in the present state of pathology, would require a very extended discussion for their complete elucidation: it will be desirable, therefore, here rather to give the opinions of the author than to enter into any abstract speculations concerning them. At the same time it will be observed, that many of the views brought forward as peculiar to the French eclectic school are sufficiently familiar to British pathologists, although, from the ignorance of M. D'Amador of the writings of our countrymen, he has been led into the error of considering them as of very recent date.

"One of the errors of the exclusive anatomico-pathological school was the rejection of internal predisposing causes, and the exclusive study of occasional causes. The insufficiency of the alterations of the solids in the theory of disease has necessarily led to the study of the liquids; and thence has arisen the whole system of the anatomical humoral pathology (*l'humorisme anatomique*). Each liquid globule lives, assimilates nourishment, secretes, and is susceptible of passing through every degree of organization; and it is upon this fact that the modern system of humoralism is really based. Now, the condition of a single globule may communicate itself to all those which circulate with it, and the participation of all the molecules in a diseased modification experienced by one only, or a small number, constitutes in fact a diathesis. This general condition, or diathesis, is revealed by pathological anatomy and by organic chemistry. What is better known than the great accumulations of serous, fatty, or lymphous depositions, effused into the cavities and infiltrated in the tissues, and bathing, as it were, every organ of the body; or than those primitive *cachexies*, as recognized by the ancients and confirmed by the investigations of the moderns; cachexies really *essential*, for the morbid alteration passes directly from the forces to the liquids, leaving the solids unaffected?" . . . . "The quantity alone of the liquids, either by augmentation or by diminution, may constitute a disease. Observers of all ages and of every school have admitted the existence of plethora,—that is, the superabundance of the sanguineous fluid,—as a fact established by experience; while, at the present day, the liquids take so essential a place in the theories of pathological anatomy, that the most marked and distinctive characters of almost every alteration of the solids is now attributed to them. It is pretty generally considered that cancerous matter is but the production of a morbid secretion, an opinion first announced by M. Cruveilhier; and the same view is taken of tubercle. The eclectic school has deprived the organization of that property with which the school of Laennec had invested it, of creating everywhere new tissues, the type of which does not exist in the normal organization. Medullary sarcoma, melanosis, cirrhosis, &c. were, according to Laennec, so many tissues of new formation. According to the recent views of organic science, these products are to be regarded rather as altered secretions; but it will always remain to be ascertained whether these morbid secretions may or may not have their analogues among the normal secretions. This view has acquired such importance that M. Andral makes it the foundation of his anatomico-pathological classifications. According to him, every degeneration of the solids resolves itself, in tracing the origin and mechanism of its formation, into an alteration of the circulation, of nutrition, or of secretion; that is, into an alteration of the liquids." (p. 449.)

According to the eclectic school, then, the organic tissues are in themselves incapable of degeneration. They are only liable to atrophy or hypertrophy,—to a diminution or increase in their nutrition. It follows that every alteration of texture arises, without exception, from the deposition of secreted matters in the meshes of the cellular tissue. These secretions accumulate, become developed and organized; thus gradually encroaching upon the normal tissues, and ultimately altogether destroying them by inducing a state of atrophy. It results from these speculations, which profess to be founded on the facts of pathological anatomy, that there are diseases which consist in general conditions, as they are termed by the author; conditions of disease existing throughout the whole body, before any localization of the morbid process shall have determined the explosion of the disease upon any particular organ or tissue.

The second question proposes for consideration the influence of these diatheses, viewed as occasional causes in the production of organic alterations. Bichât was inclined to suppose that each tissue has its own peculiar diathesis; but a more comprehensive view fully establishes the fallacy of this opinion. It is indisputable that certain diseased actions manifest themselves more commonly in certain organs or tissues than in others: thus tubercle, though found in every part of the body, is far more frequently deposited in the lungs than in any other site; carcinoma in the mamma and uterus, &c. M. D'Amador says, that it would appear that certain lesions are more prone to occur in one or other organ, according to the more or less intimate relation of the diathesis giving rise to them with the intimate structure of the parts. This may possibly be an expression of the fact; but it affords no explanation of it, no more than the substitution of one arbitrary symbol for another, in an algebraical formula, would lead to the detection of the unknown quantity which it is intended to represent. There is little in this part of the treatise which need detain us, since it consists chiefly in the exposition of what is already well known. The following are the conclusions at which the author arrives:

"Diatheses, then, should be considered as peculiar states, either of the solids or especially of the liquids, and, above all, of the blood, constituting a kind of vitiated nutrition, sometimes obscure and latent, sometimes characterized by its effects upon various parts of the economy. Hence it is that local treatment of these affections is never sufficient to eradicate them from the system; that, when they disappear from one part, they reappear at another. It is by modifications of the general influences alone that the constitutional state can be changed and the disease destroyed." . . . "We conclude, therefore, that, if the knowledge of the seat of the affection is advantageous, the knowledge of the diathesis is indispensable; for, possessed of this last, there is a much better prospect of the removal of organic disease already existing, and of its prevention where it has not already been formed." (p. 459.)

The third question, referring to the influence of inflammation as a diathesis and as an exciting cause of change of structure, we pass over, contenting ourselves with quoting the conclusion, of which, indeed, the remarks preceding it are but the amplification; viz. that inflammation is to be looked upon only as a particular diathesis, and is by no means to be considered as the sole and universal cause of diseases.

Previously to bringing this notice to a conclusion, we must make a few observations upon the Essay of M. Saucerotte. Like that to which we have already devoted so much attention, it is divided into two sections. The first section comprehends a brief sketch of the state of medicine, considered as a science, previous to the epoch of Morgagni; and treats of the influence which pathological anatomy has had upon the knowledge of the seat, nature, and treatment of diseases; its influence upon theory, and the philosophical spirit developed in consequence of its cultivation; the methods employed in the study of pathological anatomy, in relation to the influence which these methods have exercised upon medicine, and the errors introduced by it into the science. The second part treats of the influence which pathological anatomy has exercised on the knowledge of diseases considered in detail. These are grouped together under the several heads of diseases of the cerebro-spinal apparatus, diseases of the circulatory system, of the respiratory system, of the digestive tube and its dependencies, and general diseases affecting the whole frame or certain tissues only. Some remarks upon the influence of pathological anatomy on certain specialities of pathology, and its application in general to the medical sciences, follow; and a brief retrospect concludes the essay. M. Saucerotte, notwithstanding the tenor of the observations which we have previously quoted from him, is a localist, as the following extract sufficiently evinces: "To know a disease," he observes, "is to know its seat and its nature. We can no more form a conception of any diseased affection without a seat, than we can of digestion without the stomach, or of vision without the eye." He would also seem to be a decided advocate of the organic pathology; for he asks, "Is it not, in effect, in the organs that it (disease) exists; and, if we know not the organs diseased, how can we know the diseases?" Nevertheless, in the subsequent discussion of these principles, and in attempting to illustrate them by an appeal to observed facts, the author is obliged, in part at least, to swerve from his position. Some of these passages we had marked for quotation and comparison with the opinions expressed by M. D'Amador; but the length to which this notice has already extended compels us to forego our intention. Our opinion of the merits of these dissertations may be gathered from what we have already had occasion to observe respecting them; but it would scarcely be just to M. Saucerotte, were we not to add that he seems to have spared no pains in the investigation of his subject; and, although his treatise perhaps is not equal, as a philosophical production, to that of M. D'Amador, it yet possesses very meritorious qualities, affords a good view of the present state of our knowledge, and evinces an acquaintance with the medical literature of other countries beside his own; a quality in which, we regret to say, the essay of M. D'Amador is deficient.

